# Unusual giant plunging sublingual epidermoid cyst: A case report and review of literature

**DOI:** 10.1002/ccr3.9067

**Published:** 2024-06-11

**Authors:** Mohamad Safwan, Aditya Amit Godbole, Arens Jean Ricardo Médéus, Oxiris Yexalén García‐González, Vivek Sanker, Polasu Sri Satya Sai Prashanth, Tirth Dave

**Affiliations:** ^1^ Department of General and Minimal Access Surgery KIMS health Hospital Trivandrum Kerala India; ^2^ Team Erevnites Trivandrum India; ^3^ Bharati Vidyapeeth (Deemed to be University) Medical College Pune India; ^4^ Department of General Surgery Hospital of the State University of Haiti Port‐au‐Prince Haiti; ^5^ Surgical Research and Global Education Lab Port‐au‐Prince Haiti; ^6^ Monterrey Institute of Technology and Higher Education Monterrey Mexico; ^7^ Monterrey Institute of Technology and Higher Education Guadalajara Mexico; ^8^ Stanford University Stanford California USA; ^9^ Ramaiah Medical College Bangalore India; ^10^ Bukovinian State Medical University Chernivtsi Ukraine

**Keywords:** cysts, head and neck, intra‐Oral approach, sublingual epidermoid

## Abstract

**Key Clinical Message:**

When treating a painless or asymptomatic mass in the submental or floor of the mouth, sublingual epidermoid cyst should be considered. Despite its irregularity, preventing malignant transformation is essential for a successful outcome.

**Abstract:**

Dermoid and epidermoid cysts are rarely found in the head and neck region. They account for less than 0.01% of all oral cavity cysts. This is a rare case of a sublingual epidermoid cyst of the oral cavity in a 25‐year‐old male. The patient presented with a painless sublingual swelling for a duration of 1 month. The clinical examination revealed a non‐tender swelling in the sublingual region extending to the submental triangle. Magnetic resonance imaging confirmed a 6.2 × 7.7 × 3.2 cm cystic lesion in the sublingual space. Fine needle aspiration cytology confirmed dermoid cyst contents. Intra‐oral surgical excision under general anesthesia was performed successfully. Histopathological analysis revealed that the cyst wall was lined by stratified squamous epithelium. The presence of a prominent granular layer and keratin flakes confirmed the diagnosis of an epidermoid cyst. Postoperative recovery was good, and no recurrence was observed during follow‐up. This case emphasizes the infrequent and unusual presentation of a case of a giant plunging sublingual epidermoid cyst and promotes awareness and potential studies in the enhancement of patient care in this area.

## Graphical abstract

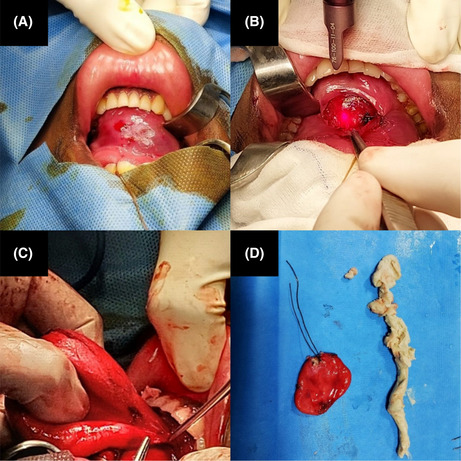

## 1 BACKGROUND

Epidermoid cysts account for approximately 1.6%–6.9% of all cysts in the head–neck‐face region. Cystic spaces lined solely by epithelium is a characteristic feature of epidermoid cysts.^1^ Depending on their size, an epidermoid cyst in the floor of the mouth can result in breathing, speaking, and swallowing difficulties.^2^ Guided by cyst size, an intra‐oral or extra‐oral surgical excision is the primary treatment modality.^3^ Our case emphasizes the rarity of oral epidermoid cysts and highlights successful surgical management with excellent functional and esthetic outcomes. It contributes valuable insights into their clinical presentation, diagnosis, and treatment.

## 2 CASE REPORT

### 2.1 Case history

**FIGURE 1:**
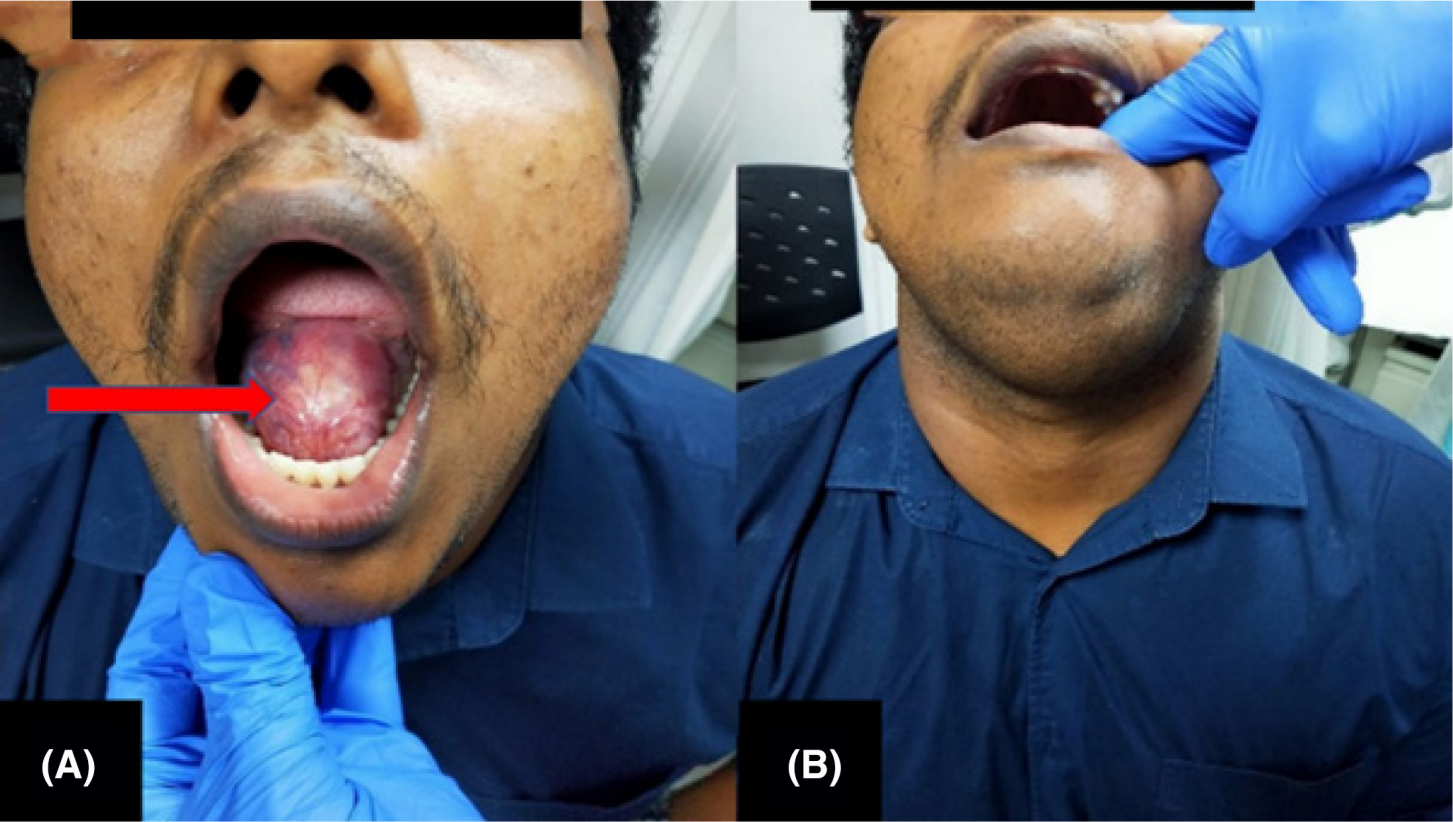
(A and B) Clinical examination showing a well‐circumscribed swelling over the sublingual region and its extension to the submental region.

A 25‐year‐old gentleman presented with a complaint of painless swelling over the floor of the mouth for a duration of 1 month which is gradually progressing in size. There was no history of fever, difficulty in chewing, or dysphagia. There was no prior history of any surgery or trauma to the neck. On clinical examination showed a well‐circumscribed, non‐tender, non‐trans‐illuminating, bi‐digitally palpable, and firm swelling that is present over the floor of the mouth, and extending proximally to the submental triangle (Figure 1A,B). No cervical lymphadenopathy was noted. Systemic examination was unremarkable.

### 2.2 Methods

**FIGURE 2:**
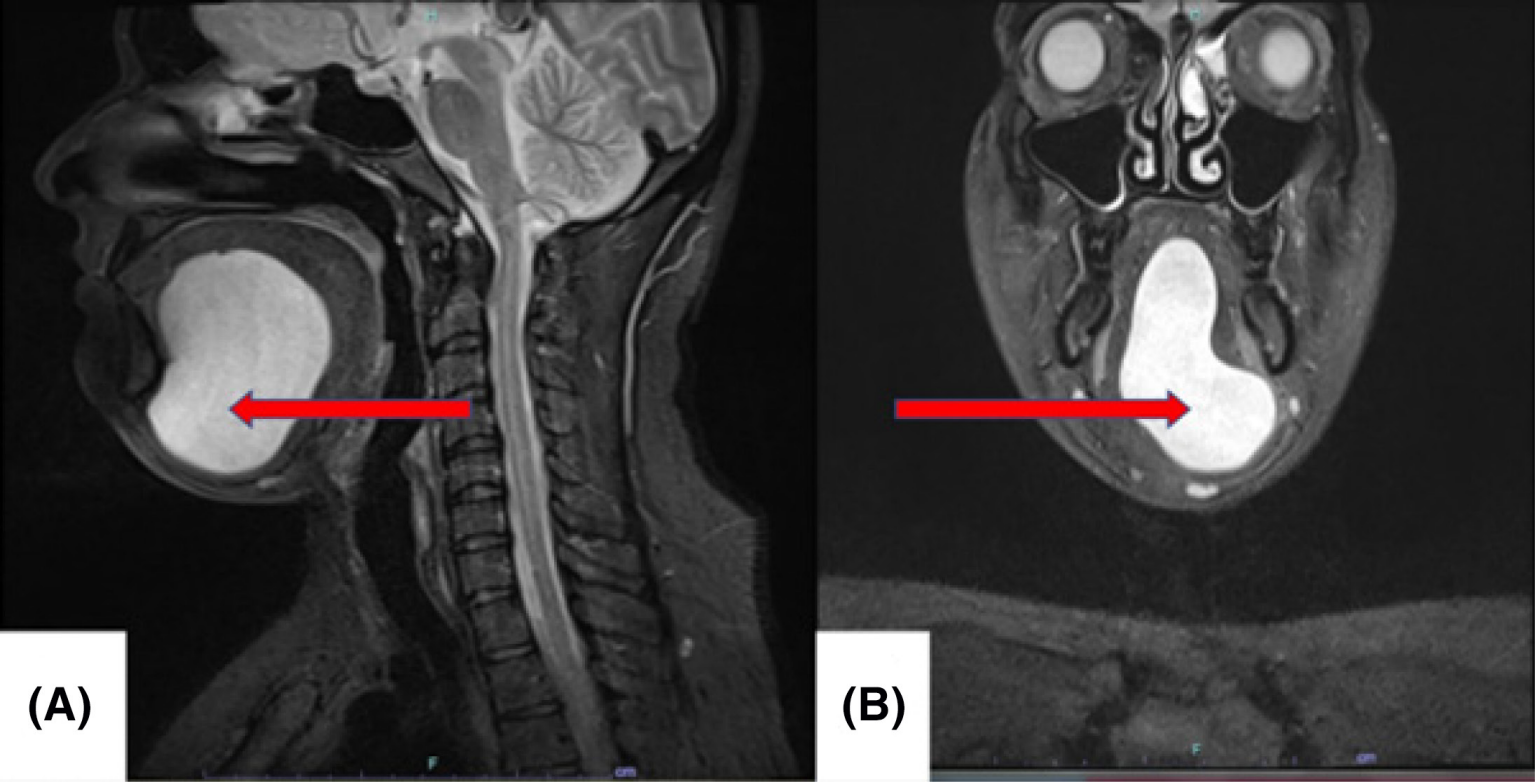
(A and B) T‐2 weighted MRI sagittal section and axial view showing the extent of the swelling.

T‐2 weighted Magnetic resonance imaging (MRI) showed a well‐circumscribed oval unilocular midline cystic lesion measuring 6.2 × 7.7 × 3.2 cm (CC × AP × TR) in the sublingual space. The cyst was splaying the genioglossus and mylohyoid muscles to either side. On the left, the lesion extended beyond the confines of sublingual space into the submental triangle through a defect in the mid‐third of thinned mylohyoid (Figure 2A,B). Results of fine needle aspiration cytology of cyst revealed pultaceous material that was compatible with contents of dermoid cyst.

**FIGURE 3:**
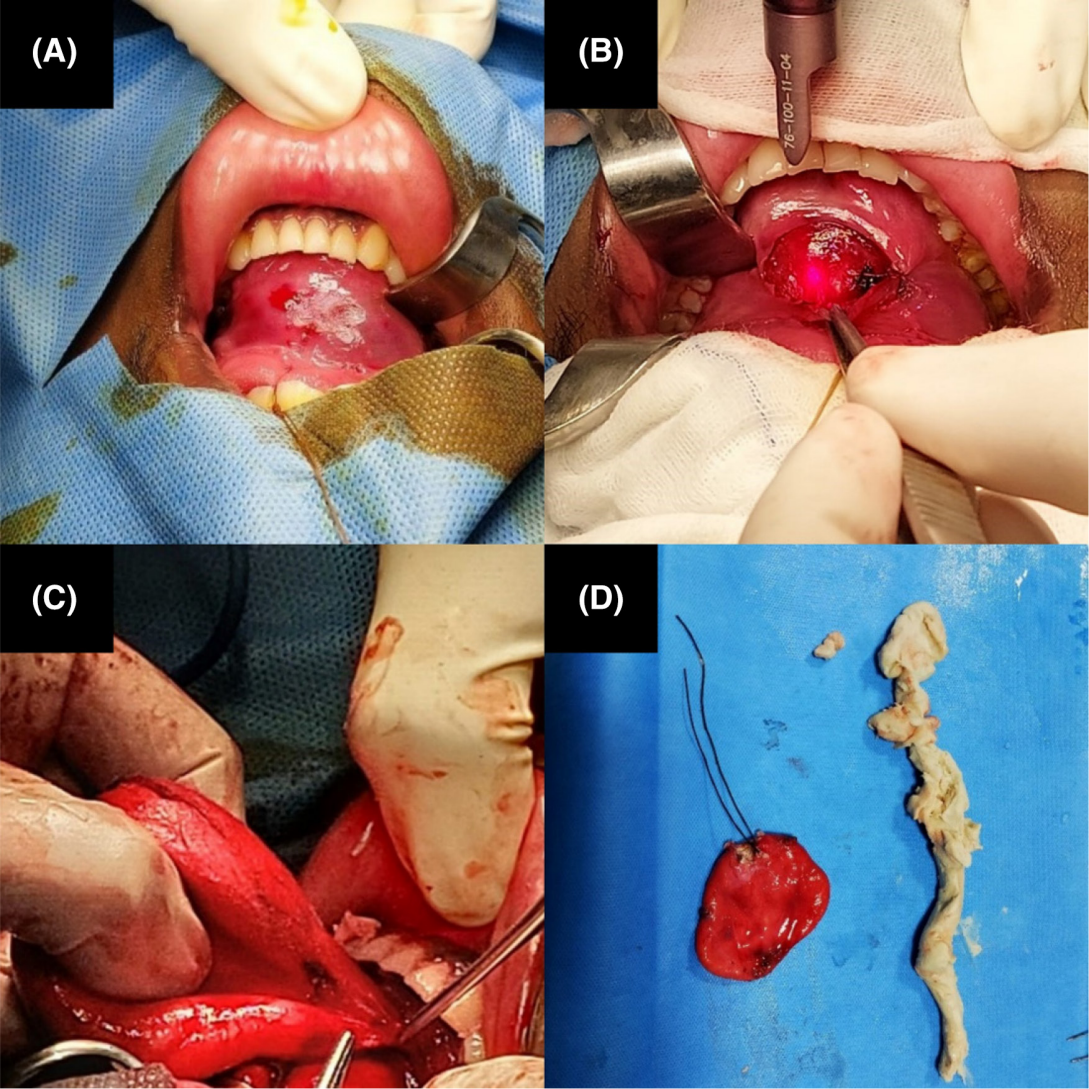
(A) Intra‐oral approach for excision of the cyst, (B) Transverse incision over the mucosa above the swelling using Co_2_ laser, (C) Extirpation of the cyst wall, (D) Excised cyst specimen with pultaceous content.

Excision of the cyst by an intra‐oral approach under general anesthesia was planned. A transverse incision was made with a CO_2_ laser scalpel on the mucosa overlying the swelling. Dissection was carried out after creating a submucosal plane around the swelling by traditional method. Bilateral submandibular ducts, their openings, and the lingual nerve were identified and preserved. Vicryl sutures were tied and the sac was opened. Cystic swelling decompression was done to facilitate extirpation of cyst wall revealing thick whitish pultaceous material. The Cyst wall was excised from the floor of the mouth. A saline and betadine wash was followed by the closure of the incision with interrupted 3–0 catgut sutures after achieving hemostasis. (Figure 3A–D) No surgical drain was placed at the site. The post‐operative period was uneventful and the patient was discharged on day 2 having reported no complications.

### 2.3 Conclusion and results

**FIGURE 4:**
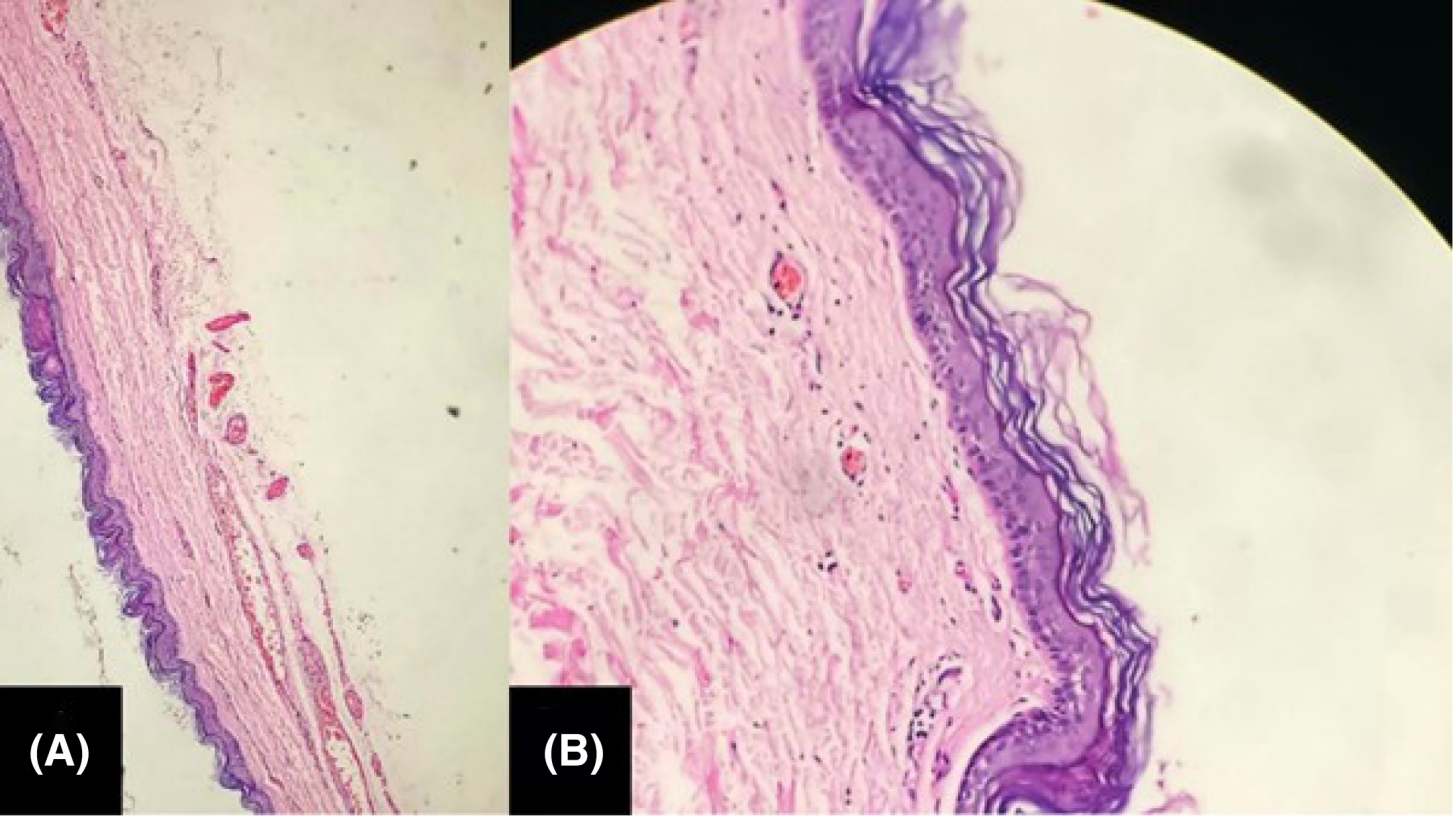
(A and B) Histopathological examination of specimen ([A] at 100× magnification and [B] at 200× magnification). Cyst wall lined by stratified squamous epithelium with a prominent granulosa layer. Adnexal structures are absent.

Normal wound healing and no complications were reported during a follow up after 1 week, 6 weeks, and 6 months. The histopathological microscopic examination of the excised cyst (Figure 4), stained using a hematoxylin and eosin stain, revealed a cyst wall with a stratified squamous epithelium lining. It had a prominent granulosa layer. Cyst contained keratin flakes. Focally, the wall showed fibrosis with dense lymphocytic infiltration and capillary proliferation. No skin appendages were noted. The surgical site was healthy and no recurrence was found on follow‐up.

## 3 DISCUSSION

Sublingual epidermoid cysts are rare and represent approximately 0.01% of oral and maxillofacial cystic lesions.^4^, ^5^ The sublingual cyst can be histopathologically classified into three types. The first type, epidermoid cysts, is characterized by the presence of an epithelial lining without skin appendages. The second type, dermoid cysts, includes skin appendages, such as hair, follicles, and sebaceous glands within the cystic cavity. Teratoid cysts contain skin appendages and encompass mesodermal elements such as bone, muscle, or respiratory system tissue.^6^, ^7^ The etiology of these cysts remains uncertain congenital dermoid and epidermoid cysts are believed to result from embryological accidents that occur during early development.^8^ It is the ectodermal differentiation or the epithelial cells entrapped during midline closure of the branchial arches that are thought to contribute to their formation.^7^, ^8^ Acquired cysts are known to originate from either traumatic or iatrogenic inclusion of epithelial cells or from the blockage of sebaceous gland ducts.^9^

Clinical examination revealed a well‐circumscribed swelling in the submental triangle, which gradually increased in size. Importantly, the absence of pain, fever, or functional difficulties in chewing or swallowing helped to distinguish this case from other differential diagnoses. Epidermoid cysts typically present as an asymptomatic mass that gradually increases in size.^6^ However, in some cases, patients may present signs of compression, such as dysphagia, dyspnea, and dysphonia. Certain cases report a presentation with a “double chin” due to further growth of the cyst in an inferior direction.^10^, ^11^, ^12^

Accurate diagnosis and optimal preoperative planning are essential for the management of sublingual epidermoid cysts. Imaging modalities, such as ultrasound, CT, and MRI, play a crucial role in determining the location and characteristics of cysts.^10^, ^11^ Ultrasonography reveals solid and cystic structures within a heterogeneous mass, while CT scans display unilocular masses with thin walls filled with hypoattenuating fluid and fat nodules, presenting a characteristic “sack‐of‐marbles” appearance.^10^, ^13^ MRI accurately delineated the size, location, and anatomical relationships of the lesion and fine needle aspiration cytology is a safe, economical, and dependable technique that can provide valuable information for the analysis of sublingual lesions.^7^ This information aided in surgical planning and guided the choice of an intraoral approach for cyst excision.^10^, ^13^ In this case, the location could be determined by MRI. The cyst extended over the genioglossus and mylohyoid muscles and protruded into the sublingual space on the left side, indicating a plunging epidermoid cyst.

Surgical excision is the preferred treatment modality, with the aim of complete removal of the cyst wall while avoiding rupture to prevent postoperative inflammation.^10^, ^13^, ^14^ The use of a CO2 laser is an alternative to conventional surgery, enabling precise tissue dissection and minimizing trauma, as was performed in this case.^14^ Recurrence rates are low after total surgical excision.^10^ Although rare, a few cases have reported a malignant transformation to squamous cell carcinoma or basal cell carcinoma.^10^, ^13^

Sublingual plunging epidermoid cysts may pose a diagnostic and therapeutic challenge. Early recognition, aided by clinical examination and imaging techniques such as MRI, is crucial for accurate diagnosis and appropriate management. Surgical excision remains the mainstay of treatment. While the approach depends on size and location of the swelling, meticulous dissection is essential to preserve vital structures and achieve optimal outcomes.

### 3.1 Review of literature

Our search approach involved crafting a comprehensive search string incorporating relevant keywords and Boolean operators. We aimed to capture literature from both Google Scholar and PubMed databases. Specifically, our search terms encompassed variations related to epidermoid cysts, dermoid cysts, and sublingual cysts, considering their anatomical localization within the oral cavity, including the floor of the mouth, submandibular, and sublingual regions. Additionally, we included terms reflecting diverse aspects of the articles, such as imaging characteristics, case studies, management strategies, pediatric cases, diagnostic approaches, treatment modalities, and literature reviews. The following search strategy was implemented: (“epidermoid cyst” OR “dermoid cyst” OR “sublingual cyst”) AND (“floor of the mouth” OR “oral cavity” OR “submandibular” OR “sublingual”) AND (“imaging features” OR “case report” OR “management” OR “pediatric” OR “diagnosis” OR “treatment”) AND (“literature review” OR “review of cases” OR “report of cases” OR “narrative review”).

We identified 40 case reports and case series on sublingual epidermoid cyst which have been summarized in Table 1.^4^, ^5^, ^7^, ^9^, ^10^, ^11^, ^12^, ^13^, ^15^, ^16^, ^17^, ^18^, ^19^, ^20^, ^21^, ^22^, ^23^, ^24^, ^25^, ^26^, ^27^, ^28^, ^29^, ^30^, ^31^, ^32^, ^33^, ^34^, ^35^, ^36^, ^37^, ^38^, ^39^, ^40^, ^41^, ^42^, ^43^, ^44^, ^45^, ^46^ Patient demographics revealed a varied age range from infants to 77 years, with predominant being male. Common clinical presentations included slow‐growing, painless, non‐fluctuant swellings in the floor of the mouth. Less common symptoms encompassed difficulties in speech, swallowing, breathing, occasional tenderness, and asymptomatic cases. Rarely, patients experienced painful non‐fluctuant swellings or asymptomatic sublingual swellings. The dimensions of cysts varied across studies, with some reaching sizes up to 10 cm × 8 cm. Surgical excision, primarily via intraoral approaches, was the prevailing management strategy, supplemented by additional procedures such as marsupialization, excision with intact capsule, sublingual gland excision, and intralesional steroid injection. A few cases opted for conservative surgical excision. The overall prognosis was favorable, with a low recurrence rate and most patients experiencing an excellent recovery, marked by a swift postoperative period, as reported in follow‐up periods ranging from 6 months to 10 years.

**TABLE 1:** List of previously published case reports.

Author	Year	Age (Years)	Gender	Clinical Presentation	Clinical Features of the Swelling	Dimensions of the cyst (mm)	Management	Follow‐up
Basla et al.^13^	2023	17	Male	A painless swelling in the floor of the mouth for 3 months. The swelling had gradually increased in size and was now causing some difficulty with speech.	Painless non‐fluctuant swelling	50 × 40 × 45	Surgical excision	No recurrence
Rai et al.^15^	2023	25	Male	Painless swelling in the floor of the mouth for 6 months. The swelling had gradually increased in size and was now causing some difficulty with speech. There was also a 1 cm, mobile, non‐tender lymph node in the left cervical chain	Painless non‐fluctuant swelling	57 × 63 × 24	Surgical excision	No recurrence
Naik et al.^16^	2023	55	Male	A painless, fluctuant swelling in the floor of the mouth that had been present for 6 months. The swelling had gradually increased in size and was now causing some difficulty with speech and swallowing.	Painless fluctuant swelling	80 × 65 × 40	Surgical excision	No recurrence
Erol & Laçin^17^	2022	55	Male	Painless, fluctuant swelling in the floor of the mouth for 6 months	Painless fluctuant swelling	50 × 50	Surgical excision	No recurrence
Sakat et al.^18^	2021	22	Male	Submental mass with pain in the throat, difficulty in chewing and swallowing solid food, submental swelling, difficulty in breathing and swallowing, decreased tongue movements and snoring	Painful non‐fluctuant swelling	75 × 65	Surgical excision	No recurrence
Sakat et al.^18^	2021	23	Female	Difficulty in chewing and swallowing solid food	Painless fluctuant swelling	40 × 40 × 50	Surgical excision	No recurrence
Sakat et al.^18^	2021	28	Male	Dyspnea, shortness of breath and a painless, growing mass in the submental region	Painless fluctuant swelling	50 × 50 × 60	Surgical excision	No recurrence
Hashimoto et al.^19^	2021	59	Male	Asymptomatic unilocular radiolucent area at his anterior maxilla	Painless non‐fluctuant swelling	15 × 20	Surgical excision	No recurrence
Klibngern & Pornchaisakuldee^4^	2020	22	Female	Slow‐growing mass at the submandibular area and swelling in the floor of mouth	Painless fluctuant swelling	65 × 32 × 25	Surgical excision	No recurrence
Misch et al.^20^	2020	5 patients of epidermoid cyst with mean age 2.4 years	2 Females, 3 Males	Slow‐growing, painless, non‐fluctuant, firm mass in the sublingual region	Painless non‐fluctuant swelling	Multiple cysts of varying sizes	Surgical excision	No recurrence
Thibouw & Schein^21^	2020	73	Female	Difficulty in speaking	Painless non‐fluctuant swelling	70 × 40 × 35	Surgical excision	No recurrence
Kumari et al.^22^	2018	6	Female	Swelling in the floor of the mouth beneath the tongue, asymptomatic	Asymptomatic	40 × 50	Surgical excision	No Recurrence
Findik et al.^7^	2017	10	Male	Slow‐growing, painless, non‐fluctuant mass in the floor of the mouth for 6 months.	Painless non‐fluctuant swelling	30 × 40 × 40	Surgical excision	No Recurrence
Sahoo et al.^10^	2017	55	Female	Gradual increasing painless swelling of the floor of the mouth and submental region under the tongue and beneath the chin with difficulty in speech and swallowing for 6 months of duration.	Painless fluctuant swelling	Data not provided	Surgical excision	No Recurrence
Nishar et al.^23^	2016	43	Male	Giant, asymptomatic sublingual swelling	Asymptomatic	100 × 80	Surgical excision	No Recurrence
Reddy et al.^24^	2016	19	Female	Enlarging, occasionally tender right‐sided neck mass for 1 year	Painful fluctuant swelling	40 × 30 × 30	Surgical excision	No Recurrence
Reddy et al.^24^	2016	10	Not Given	Nontender swelling on the right side of the floor of mouth	Painless fluctuant swelling	32 × 29 × 28	Surgical excision	No Recurrence
Gulati et al.^9^	2015	16	Male	Slow‐growing, painless swelling in the left neck region for 3 months.	Painless fluctuant swelling	62 × 60 × 57	Surgical excision	No Recurrence
Yoshida et al.^25^	2014	39	Male	Progressive left submandibular swelling for 3 months.	Painless fluctuant swelling	95 × 70 × 50	Surgical excision	No Recurrence
Oginni et al.^26^	2014	26‐days‐old infant	Male	Sublingual swelling present from birth.	Asymptomatic	40 × 30	Surgical excision	No Recurrence
Baliga et al.^27^	2014	26	Female	Large sublingual swelling causing speech and swallowing difficulties	Painless non‐fluctuant swelling	30 × 30	Surgical excision	No Recurrence
Anderson & Stassen^28^	2014	77	Female	Large floor‐of‐mouth swelling	Painless non‐fluctuant swelling	80 × 20 × 25	Surgical excision	No recurrence
Dutta et al.^29^	2013	2–60 (mean age 30)	5 Females, 23 Males	Submandibular region (5), pinna (5), sublingual region (1), periorbital (6), suprasternal (6), along the anterior border of sternocleidomastoid (1) and glabella (3), along with an iatrogenic implantation epidermoid cyst in a tracheostomy scar.	Painless fluctuant swelling	75 × 60 × 45	Surgical excision	No recurrence
Kudoh et al.^30^	2013	69	Male	Mobile, elastic, relatively soft mass without tenderness in the right submandibular	Painless fluctuant swelling	40 × 30 × 25	Surgical excision	No recurrence
Assaf et al.^12^	2012	65	Male	Gradually enlarging swelling in the floor of the mouth for 10 years	Painless fluctuant swelling	Data not provided	Surgical excision	No recurrence
Saito et al.^31^	2012	31	Male	Difficulty of swallowing and deviation of the tongue toward the posterior wall of the oropharynx.	Painless non‐fluctuant swelling	60–90 (diameter)	Surgical excision	No Recurrence
Saito et al.^31^	2012	25	Female	Swelling of the floor of oral cavity and difficulty in breathing when lying in supine position	Painless non‐fluctuant swelling	60–90 (diameter)	Surgical excision	No recurrence
Verma et al.^32^	2012	16	Female	Mass in sublingual region	Asymptomatic	70 × 50 × 45	Surgical excision	No recurrence
Tsirevelou et al.^33^	2009	45	Male	Soft, painless, movable and touchable intraoral swelling for 10 months.	Painless non‐fluctuant swelling	35 × 35	Surgical excision	No recurrence
Tsirevelou et al.^33^	2009	35	Female	Left‐sided neck swelling and an intraoral swelling for 6 months. Dysphagia, dysarthria and dyspnoea on exertion	Painless fluctuant swelling	55	Surgical excision	No recurrence
Patil et al.^5^	2009	28	Male	Well circumscribed, distinct, dome shaped sessile midline swelling extending	Painless fluctuant swelling	30 × 200 × 20	Surgical excision	No Recurrence
Bhatt et al.^34^	2008	64	Female	Swelling in the floor of the mouth	Painless non‐fluctuant swelling	Data not provided	Surgical excision	No recurrence
Pereira et al.^35^	2008	60	Female	Bulging in the belly of the tongue.	Painless non‐fluctuant swelling	8 × 4 × 5	Surgical excision	No recurrence
Kandogan et al.^36^	2007	11	Male	Mass in the oral cavity, difficulty chewing and swallowing of solid foods	Painless non‐fluctuant swelling	40 × 35	Surgical excision	No recurrence
Koca et al.^37^	2007	20	Male	Swelling in the floor of his mouth that was causing difficulties with speech	Painless non‐fluctuant swelling	40 × 30	Surgical excision	No recurrence
Jham et al.^11^	2007	25	Male	Large sublingual swelling	Painless non‐fluctuant swelling	50 × 50	Surgical excision	No recurrence
Yilmaz et al.^38^	2006	34	Female	Cosmetic problems, presence of swelling	Painless non‐fluctuant swelling	45 × 60 × 75	Surgical excision	No Recurrence
Yilmaz et al.^38^	2006	35	Male	Difficulty swallowing	Painless non‐fluctuant swelling	50 × 70 × 80	Surgical excision	Data not provided
Bitar & Kumar^39^	2003	17	Male	Sublingual mass pushing the tongue upward	Painless non‐fluctuant swelling	80 × 51 × 47	Surgical excision	No recurrence
De Ponte FS et al.^40^	2002	18	Male	Large swelling of oral floor.	Painless non‐fluctuant swelling	45	Surgical excision	No recurrence
Behl et al.^41^	2001	22	Male	Progressively increasing swelling of the floor of the mouth and suprahyoid neck of 18 months duration.	Painless fluctuant swelling	100 × 80	Surgical excision	No recurrence
Turetschek et al.^42^	1995	Data Not Provided	Both	Not provided	Asymptomatic	60 × 50	Surgical excision	Data not provided
Calderon & Kaplan^43^	1993	4‐days‐old	Male	Mass in tongue and sublingual space	Painless non‐fluctuant swelling	30 × 20	Surgical excision	No recurrence
Worley and Laskin^44^	1993	9	Male	Large, painless swelling of the floor of the mouth	Painless non‐fluctuant swelling	50 × 50	Surgical excision	No recurrence
Potts et al.^45^	1992	22	Female	Midline sublingual epidermoid cyst	Painless non‐fluctuant swelling	60 × 40	Surgical excision	No recurrence
Benoliel et al.^46^	1990	28	Female	Growing swelling in the left submandibular area	Painless non‐fluctuant swelling	Data not provided	Surgical excision	No recurrence

## 4 CONCLUSIONS

Sublingual epidermoid should be kept in mind when dealing with a painless or asymptomatic mass in the floor of the mouth or sub‐mental region. Despite its irregularity, maintaining vigilance against malignant transformation is critical. Therefore, early detection, precise diagnosis, and effective intervention are essential for a good functional and esthetic outcome.

## AUTHOR CONTRIBUTIONS

**Mohamad Safwan:** Conceptualization; data curation; formal analysis; funding acquisition; investigation; methodology; writing – original draft; writing – review and editing. **Aditya Amit Godbole:** Project administration; resources; software; supervision; validation; visualization; writing – original draft; writing – review and editing. **Arens Jean Ricardo Médéus:** Project administration; resources; software; supervision; validation; visualization; writing – original draft; writing – review and editing. **Oxiris Yexalén García‐González:** Project administration; resources; software; supervision; validation; visualization; writing – original draft; writing – review and editing. **Vivek Sanker:** Project administration; resources; software; supervision; validation; visualization; writing – original draft; writing – review and editing. **Polasu Sri Satya Sai Prashanth:** Resources; software; supervision; validation; visualization; writing – original draft; writing – review and editing. **Tirth Dave:** Resources; software; supervision; validation; visualization; writing – original draft; writing – review and editing.

## CONFLICT OF INTEREST STATEMENT

The authors have no conflict of interest to declare.

## ETHICAL APPROVAL

Ethical approval was not required for the case report as per the country's guidelines.

## CONSENT

Written informed consent was obtained from the patient to publish this report in accordance with the journal'spatient consent policy.

## Data Availability

The data supporting this article's findings are available from the corresponding author upon reasonable request.
